# Impact of Internet Addiction on Patient-Reported Outcomes in Chinese Children Aged 8 to 18 Years With Malignant Tumors: A Cross-Sectional Study

**DOI:** 10.7759/cureus.83038

**Published:** 2025-04-26

**Authors:** Li Lanxing, Jiang Xiaoping, Lin Xin, Xiao Dan

**Affiliations:** 1 Department of Nursing, Children's Hospital of Chongqing Medical University, Chongqing, CHN; 2 Department of Hematology and Oncology, Children's Hospital of Chongqing Medical University, Chongqing, CHN; 3 Department of Orthopedics, Children's Hospital of Chongqing Medical University, Chongqing, CHN

**Keywords:** child and adolescent, internet addiction, malignant tumors, patient-reported outcome, psychiatry & mental health

## Abstract

Background

In the context of the digital era, the deep integration of the Internet and the medical field has shown two sides in the treatment and rehabilitation of children with malignant tumors. However, the problem of Internet addiction (IA) behind Internet use in these children - and its potential threat to their health status - has not yet attracted widespread attention. The main objective of this study was to investigate the impact of IA on patient-reported outcomes (PROs) in Chinese children aged 8 to 18 years with malignant tumors.

Methods

From October 2023 to May 2024, a continuous sampling of 300 children aged 8 to 18 with malignant tumors was conducted at the National Clinical Medical Research Center for Child Health and Diseases in Chongqing. IA was assessed using the Internet addiction test (IAT). In contrast, the Chinese version of the Pediatric Patient-Reported Outcome Measurement Information System (C-Ped-PROMIS) was used to evaluate the PROs. The relationship between these variables was analyzed using Spearman rank correlation analysis and multiple linear regression models.

Results

Out of 300 children, 87 (29.0%) showed signs of IA, with elementary school students having a significantly lower rate than students in middle school and above (p = 0.005). IA was positively correlated with depression (r = 0.127, p < 0.05), anger (r = 0.130, p < 0.05), anxiety (r = 0.158, p < 0.01), and fatigue (r = 0.129, p < 0.05) scores. Multiple linear regression analysis showed that elevated IAT scores were significant negative predictors of depression, anger, anxiety, and fatigue (all p < 0.05).

Conclusion

Children aged 8 to 18 years with malignant tumors face a higher risk of IA, which is closely related to some self-reported outcomes. Healthcare professionals should pay attention to the Internet usage issues of children with malignant tumors, guiding them to use the Internet wisely to improve their mental health and overall well-being.

## Introduction

Internet addiction (IA) is a typical behavioral disorder of the digital age, usually referring to an individual's impulsive, uncontrolled use of the Internet without the effect of addictive substances, which in turn leads to impaired academic, occupational, and social functioning [1]. According to a survey conducted by the China Internet Network Information Center (CNNIC), by December 2024, children and teenagers aged 6 to 19 made up 16.7% of the total internet users in China [2]. IA is closely related not only to academic impairment and family relationship conflicts, but also to psychological symptoms such as anxiety and depression, as well as physiological issues like musculoskeletal disorders [3-5]. Although China officially enacted legislation in January 2024 to strengthen Internet protection for minors, the issue of IA among children with malignant tumors - a high-risk group - has not received sufficient attention. The Lancet Oncology Commission estimates that between 2020 and 2050, there will be 13.7 million new cases of childhood cancer worldwide [6]. Under the pressures of medical demands and social isolation, children with malignant tumors exhibit emotional needs and behavioral patterns that significantly differ from those of healthy children [7]. With the continuous integration of the Internet and the medical field, many functional games and mobile health technologies are being applied to the disease treatment and symptom management of children with malignant tumors [8,9]. Despite the clear advantages of these technologies in disease treatment, they also inadvertently increase the opportunities and time for Internet use among these children, leading to a broadly overlooked risk of addiction. Research by Schindera et al. and Bratteteig et al. has shown that the screen exposure time of children with malignant tumors far exceeds the average daily online time of their peers in Europe [10,11]. Yet, there has been insufficient in-depth research into the hidden addiction issues and potential health problems associated with this.

Patient-reported outcomes (PROs) are direct reports by patients of their health status, functional status, and feelings about treatment [12]. The Chinese version of the Pediatric Patient-Reported Outcomes Measurement Information System (C-Ped-PROMIS) is an essential health assessment tool that can comprehensively reflect patients' physiological, psychological, and social functional status, holding significant value in the health management of children with cancer [13]. The core objective of this study was to clarify the association between IA and PROs in Chinese children aged 8 to 18 years with malignant tumors. By revealing the connection between IA and children's health outcomes, new perspectives are provided for clinical medical staff, assisting them in better understanding and managing the health issues of children with cancer, ultimately enhancing overall recovery outcomes and quality of life for these patients.

## Materials and methods

Patients

In this cross-sectional study, children with malignant tumors who were seen by the National Clinical Medical Research Center for Child Health and Diseases (Chongqing) from October 2023 to May 2024 were recruited using the consecutive sampling method. The National Clinical Medical Research Center for Child Health and Diseases (Chongqing) is a leading national-level institution dedicated to advancing child health research and clinical practice. It offers comprehensive medical services for children, including diagnosis, treatment, and follow-up care for various pediatric diseases, with a particular focus on pediatric oncology. Its research and clinical expertise make it an ideal setting for conducting research on malignant tumors in children. Inclusion criteria included: (1) diagnosis of malignant tumor confirmed by pathology or imaging; (2) age 8-18 years (according to international standards, children ≥8 years old can self-assess their health status [14]); (3) basic literacy skills and the ability to communicate effectively with the researcher; and (4) informed consent of the legal guardian and voluntary participation of the children themselves. Exclusion criteria were: (1) critical illness or protective isolation; (2) severe mental disorders (including severe cognitive disorders, severe behavioral disorders, and severe schizophrenia) that rendered them incapable of taking the test.

General information questionnaire

The baseline data of the children collected in this study included demographic characteristics and clinical information. Demographic information included age, gender, education stage, family residence, and per capita monthly family income. Clinical information mainly included: (1) tumor type, classified into five categories - leukemia, lymphoma, central nervous system (CNS) tumors, solid tumors outside the CNS, and sarcoma - based on the International Classification of Childhood Cancer (Third Edition) (ICCC-3) [15]; (2) length of time since diagnosis (from the time of initial diagnosis to the time of enrollment); and (3) body weight status, assessed by body mass index (BMI = body weight (kg)/height² (m²)), which was categorized into four grades: wasting (below the 5th percentile), standard (5th-85th percentile), overweight (85th-95th percentile), and obese (≥95th percentile), based on the BMI reference values for the gender and age group of 6-18 years in the Screening for Overweight and Obesity in School-Aged Children and Adolescents [16].

Internet addiction test (IAT)

Young's IAT was used to assess IA [17]. This study used the professionally translated Chinese version of the IAT scale. This 20-item instrument evaluates core symptoms, including preoccupation, tolerance, social withdrawal, and loss of control, using a five-point Likert scale (1 = never to 5 = always), with total scores ranging from 20 to 100. Consistent with Young's original validation study and subsequent research, a cutoff score of ≥50 was adopted to define IA [18]. The scale showed excellent reliability in our sample (Cronbach's α = 0.863).

C-Ped-PROMIS

In this study, the Chinese version of the C-Ped-PROMIS, developed by the National Institutes of Health (NIH), was used to assess the health status of children [13]. The scale comprises eight short scales focusing on fatigue, pain, anger, anxiety, depression, peer relationships, upper extremity functioning, and mobility, with a total of 64 items (6-10 items per short scale). Items within each short scale are rated on a five-point Likert scale. For all items, scores range from 0 to 4, with different anchors based on the scale type (e.g., "never" to "always" for symptom frequency, "no difficulty at all" to "unable to accomplish" for functional difficulty). The raw score of each dimension (with a response rate >50%) is calculated as the sum of item scores, then converted to a standardized score using the formula $T = \left( \frac{\text{raw score} - \text{normative mean}}{\text{normative standard deviation}} \right) \times 10 + 50$. Higher scores in symptomatic dimensions indicate more severe symptoms, while higher scores in functional dimensions suggest better functioning. The scale demonstrated good reliability, with Cronbach's α coefficients ranging from 0.885 to 0.928 for individual dimensions, and 0.941 for the overall scale.

Sample size

According to the Kendall sample size estimation method (which requires a sample size of 5-10 times the number of variables), this study's theoretically needed sample size was calculated to be 85-170 cases based on 17 variables. To ensure the reliability and validity of the study, we further considered the 20% invalid recovery rate of the questionnaires and expanded the target sample size to 102-204 cases. Finally, 300 valid questionnaires were recovered.

Statistical analysis

IBM SPSS Statistics for Windows, Version 26 (Released 2019; IBM Corp., Armonk, NY, USA), was used for data analysis. The normality of the IAT scores and scores of each short scale of the C-Ped-PROMIS was examined using the Kolmogorov-Smirnov test. The results of the Kolmogorov-Smirnov test indicated that all these variables were non-normally distributed (all p < 0.05), so they were described as median (interquartile range), and comparisons between groups were made using the Mann-Whitney U test. Count data were expressed as the number of cases and the composition ratio. Spearman's rank correlation analysis assessed correlations between variables, and correlation coefficients (r) and significance (p) were calculated. Multiple linear regression models were constructed to control for confounding factors, with C-Ped-PROMIS scores for each dimension as the dependent variable, IAT scores as the core independent variable, and covariates such as age and gender included. Covariates were excluded by a variance inflation factor (VIF < 5) before modeling, and variables were screened by the stepwise regression method (α-in = 0.05 and α-out = 0.10). All tests were considered statistically significant at p < 0.05.

Ethical approval and consent

The study received approval from the Institutional Review Board of the Children's Hospital affiliated with Chongqing Medical University, Chongqing, China (approval number: 2023-355). All patients provided written informed consent to participate in this study. All children and caregivers signed a written informed consent form and fully understood the study's benefits and risks. The authors have no conflicts of interest to declare.

## Results

General information and comparison of IA in children with different characteristics

Among the 300 children with malignant tumors, there were 160 (53.3%) aged 8 to 11 years old, and 140 (46.7%) aged 12 years old and above; 178 (59.3%) were male, and 122 (40.7%) were female; 182 (60.7%) had an educational level of primary school; 193 (64.3%) had a normal body weight status; 152 (50.7%) lived in urban areas; 115 (38.3%) had a monthly per capita family income of 3,000-5,000 yuan, and 102 (34.0%) had a monthly per capita family income of less than 3,000 yuan. Clinical data showed that there were 125 (41.7%) children with leukemia and 54 (18.0%) children with lymphoma; 90 (30.0%) had a duration since diagnosis of less than one month, 109 (36.3%) had a duration of one to six months, and 101 (33.7%) had a duration of more than six months (Table 1).

**Table 1:** Comparison of IA among study participants with different characteristics Non-IA, Non-internet addiction; IA, Internet addiction; CNS, Central nervous system; RMB, Renminbi

Variable	Item	Number	Non-IA	IA	X^2^	p
Age (years)	8-11	160 (53.3)	118 (39.3)	42 (14.0)	1.259	0.262
	≥12	140 (46.7)	95 (31.7)	45 (15.0)		
Gender	Male	178 (59.3)	122 (40.7)	56 (18.7)	1.287	0.257
	Female	122 (40.7)	91 (30.3)	31 (10.3)		
Education stage	Primary school	182 (60.7)	140 (46.7)	42 (14.0)	7.884	0.005
	Middle school and above	118 (39.3)	73 (24.3)	45 (15.0)		
Body weight status	Wasting	53 (17.7)	41 (13.7)	53 (4.0)	3.936	0.268
	Normal	193 (64.3)	139 (46.3)	193 (18.0)		
	Overweight	34 (11.3)	20 (6.7)	34 (4.7)		
	Obese	20 (6.7)	13 (4.3)	20 (2.3)		
Family residence	Urban	152 (50.7)	110 (36.7)	42 (14.0)	0.28	0.597
	Rural	148 (49.3)	103 (34.3)	45 (15.0)		
Per capita monthly family income (RMB)	<3,000	102 (34.0)	72 (24.0)	30 (10.0)	0.014	0.993
	3,000-5,000	115 (38.3)	82 (27.3)	33 (11.0)		
	>5,000	83 (27.7)	59 (19.7)	24 (8.0)		
Tumor type	Leukemia	125 (41.7)	88 (29.3)	37 (12.3)	1.466	0.833
	Lymphoma	54 (18.0)	38 (12.7)	16 (5.3)		
	Sarcoma	35 (11.7)	24 (8.0)	11 (3.7)		
	CNS	26 (8.7)	17 (5.7)	9 (3.0)		
	Other types	60 (20.0)	46 (15.3)	14 (4.7)		
Length of time since diagnosis (months)	<1	90 (30.0)	64 (21.3)	26 (8.7)	2.014	0.365
	1-6	109 (36.3)	82 (27.3)	27 (9.0)		
	>6	101 (33.7)	67 (22.3)	34 (11.4)		

Among the 300 included children with malignant tumors, 87 (29.0%) had IA (Table 2). The results of the Chi-square test showed that the IA rate among those with primary education was significantly lower than that among those with a junior high school education or above (p = 0.005), and there were no statistically significant differences in IA for the remaining general information variables (p > 0.05) (Table 1).

**Table 2:** Results of IAT total score and C-Ped-PROMIS scores for each short form The results of the C-Ped-PROMIS scores for each short form are converted into standardized scores. IAT, Internet addiction test; C-Ped-PROMIS, Chinese version of the Pediatric Patient-Reported Outcome Measurement Information System; IQR, Interquartile range

Scale	Items/Subscales	Median	IQR	Min value	Max value	Number
IAT	<50	33.0	(2.0, 40.0)	2.0	49.0	213
	≥50	54.0	(52.0, 60.0)	50.0	76.0	87
	Total	41.0	(34.0, 50.0)	21.0	76.0	300
C-Ped-PROMIS	Depression	46.6	(41.7, 56.5)	40.1	90.9	300
	Anger	46.9	(39.9, 56.1)	39.9	83.9	300
	Anxiety	49.9	(41.7, 56.4)	36.9	88.9	300
	Fatigue	48.6	(39.7, 56.5)	39.4	85.4	300
	Pain	48.4	(39.3, 57.6)	39.3	77.4	300
	Peer relationships	50.6	(43.6, 56.2)	23.8	68.9	300
	Physical function - mobility	47.2	(43.1, 54.6)	39.8	92.4	300
	Physical function - upper extremity	45.0	(43.2, 54.1)	43.2	101.2	300

IAT and C-Ped-PROMIS scores and comparison between groups

The results of the Mann-Whitney U-test showed that there was a significant difference in the scores of the five symptom domains - short forms of depression, anger, anxiety, fatigue, and pain - between the two groups (p < 0.05). In contrast, the scores of the three functional domains - short forms of peer relationships, physical functioning-mobility, and physical functioning-upper extremity - were not significantly different (p > 0.05) (Table 3).

**Table 3:** Comparison of C-Ped-PROMIS short form scores between IA and non-IA groups Non-IA, Non-internet addiction; IA, Internet addiction; M(IQR), Median (interquartile range); C-Ped-PROMIS, Chinese version of the Pediatric Patient-Reported Outcome Measurement Information System

Variable	Non-IA	IA	Z	p
Depression	46.6 (41.7, 54.8)	49.9 (43.3, 61.4)	-2.25	0.024
Anger	46.9 (40.0, 53.8)	49.2 (42.2, 58.4)	-2.014	0.044
Anxiety	48.2 (40.1, 56.4)	51.5 (45.0, 59.6)	-2.519	0.012
Fatigue	47.3 (39.4, 55.2)	52.5 (42.0, 59.1)	-2.46	0.014
Pain	48.4 (39.3, 57.6)	51.5 (43.8, 51.5)	-2.379	0.017
Peer relationships	50.6 (43.6, 57.7)	49.2 (43.6, 54.8)	-0.799	0.424
Physical function - mobility	46.4 (41.4, 54.6)	48.0 (43.1, 56.2)	-1.707	0.088
Physical function - upper extremity	45.0 (43.2, 53.2)	45.0 (43.2, 55.9)	-0.426	0.670

Correlation analysis

**Figure 1:**
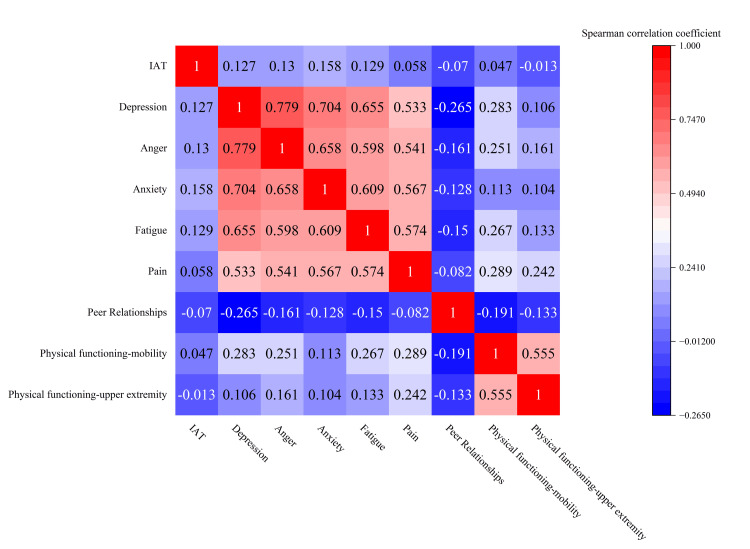
Heatmap of correlation between IA and C-Ped-PROMIS scores on each short form IAT, Internet addiction test; C-Ped-PROMIS, Chinese version of the Pediatric Patient-Reported Outcome Measurement Information System

Spearman's correlation analysis revealed that IAT was positively correlated with depression (r = 0.127, p < 0.05), anger (r = 0.130, p < 0.05), anxiety (r = 0.158, p < 0.01), and fatigue (r = 0.129, p < 0.05) scores. However, IA was not significantly correlated with pain (r = 0.058, p > 0.05), peer relationships (r = -0.07, p > 0.05), physical functioning-mobility (r = 0.047, p > 0.05), or physical functioning-upper extremity scores (r = -0.013, p > 0.05) (Table 4 and Figure 1).

**Table 4:** Correlation analysis between IAT and C-Ped-PROMIS short form scores IAT, Internet addiction test; C-Ped-PROMIS, Chinese version of the Pediatric Patient-Reported Outcome Measurement Information System

Variables	IAT	Depression	Anger	Anxiety	Fatigue	Pain	Peer relationships	Physical functioning - mobility	Physical functioning - upper extremity
IAT	1								
Depression	0.127	1							
Anger	0.130	0.779	1						
Anxiety	0.158	0.704	0.658	1					
Fatigue	0.129	0.655	0.598	0.609	1				
Pain	0.058	0.533	0.541	0.567	0.574	1			
Peer relationships	-0.07	-0.265	-0.161	-0.128	-0.150	-0.082	1		
Physical functioning - mobility	0.047	0.283	0.251	0.113	0.267	0.289	-0.191	1	
Physical functioning - upper extremity	-0.013	0.106	0.161	0.104	0.133	0.242	-0.133	0.555	1

Multiple linear regression analysis

To explore the predictive role of IAT scores and demographic variables on psychosocial functioning (depression, anger, anxiety, and fatigue) as assessed by the C-Ped-PROMIS, four multiple linear regression models were constructed. The results of the regression analyses showed that the coefficient of determination (r²) was 0.093, and the adjusted coefficient of determination (adjusted r²) was 0.064, with an F-statistic of 3.289 (p < 0.001) in the model with depression as the dependent variable. The r² and adjusted r² in the model with anger as the dependent variable were 0.069 and 0.040, respectively, with an F-value of 2.379 (p = 0.013); in the model with anxiety as the dependent variable, r² was 0.066, adjusted r² was 0.037, and the F-value was 2.287 (p = 0.017); and in the model constructed for the fatigue dimension, r² was 0.065, adjusted r² was 0.036, and the F-value was 2.258 (p = 0.019). After controlling for confounders, including age and gender, analyses showed that higher IAT scores were associated with poorer results on all measures predicting psychosocial functioning (all p < 0.05) (Table 5).

**Table 5:** Multiple linear regression analysis of Internet addiction and PROs IAT, Internet addiction test; VIF, Variance inflation factor; PROs, Patient-reported outcomes

Dependent variable	Independent variable	B	SE	β	t	p	95% CI	Tolerance	VIF
Depression	Constant	46.011	5.096	-	9.029	<0.001	35.981-56.041	-	-
	IAT	0.158	0.052	0.172	3.061	0.002	0.057-0.260	0.990	1.010
	Per capita monthly family income	-2.169	0.793	-0.170	-2.733	0.007	-3.730 to 0.607	0.809	1.237
Anger	Constant	49.780	5.163	-	9.642	<0.001	39.619-59.941	-	-
	IAT	0.144	0.052	0.157	2.757	0.006	0.041-0.248	0.990	1.010
	Per capita monthly family income	-0.253	0.804	-0.199	-0.315	0.002	-4.116 to 0.952	0.809	1.237
Anxiety	Constant	46.186	5.170	-	8.934	<0.001	36.012-56.361	-	-
	IAT	0.162	0.052	0.176	3.094	0.002	0.059-0.266	0.990	1.010
Fatigue	Constant	43.070	5.172	-	8.328	<0.001	32.892-53.249	-	-
	IAT	0.157	0.052	0.171	2.993	0.003	0.054-0.260	0.990	1.010

## Discussion

Children with malignant tumors at high risk for IA

In this study, 87 (29.0%) of the 300 children with malignant tumors had IA, a result higher than the subjective dependence rate on the Internet among minors reported in the study of essential Internet use among minors in China (19.5%) [19]. This high rate may be related to the long-term and unique nature of the treatment process for children with malignant tumors. The average number of days per hospitalization for common malignant tumors is 12-16 days, and the treatment of intermediate and advanced stages can last up to several years, which is much higher than for other pediatric diseases [20]. During the treatment period, the symptoms of the disease and the side effects of the treatment lead to difficulties in maintaining the original habits and socialization of the child, and also make them suffer from great physical pain and psychological pressure. Network compensation theory suggests that when individuals are in a negative state in real life, they tend to fulfill their cognitive and emotional needs through network use [21]. In this study, the high prevalence of IA among children with malignant tumors (29.0%) aligns with this theory, suggesting that the social isolation and psychological distress stemming from prolonged treatment may drive these children to seek emotional support and distraction online.

In this study, the rate of IA was significantly lower among those whose educational stage was elementary school than among those who were in middle school and above (p = 0.005). This may be because children in the elementary school education stage have relatively simple psychological needs and rely more on the companionship and reassurance of parents and healthcare professionals, and their Internet use may be limited to entertainment or distraction, while children in the junior and senior grades are in adolescence and have more complex psychological needs, and may seek emotional support, social interaction, or escape from the stress of the disease through the Internet. This active compensation-seeking behavior may be closely related to the increase in IA [22]. This suggests that healthcare professionals and parents should pay enough attention to children's Internet behavior while treating their diseases and take into full consideration the cognitive and psychological development characteristics of children with malignant tumors in different age groups when guiding their Internet use.

IA and psychological functioning outcomes in children with malignant tumors

This study examined the impact of IA on PROs in children with malignant tumors. Correlation analysis showed that IA was positively correlated with depression, anger, anxiety, and fatigue (p < 0.05). Multiple linear regression analysis further confirmed that IA had a significant positive effect on depression, anger, anxiety, and fatigue in children (p < 0.05). Previous studies have shown that an individual's emotional state is significantly associated with IA [23], and there is even a bidirectional association [24]. For example, Takahiro's findings suggest that IA is closely related to “pathological social withdrawal,” and that children with IA prefer online communication, feel uncomfortable with real-life interactions, and gradually lose interest in offline socialization, leading to increased loneliness and depression [25]. On the other hand, children with IA may seek the Internet as a way of coping with negative emotions, such as anxiety and depression, and alleviate some of their anxiety and depression symptoms through Internet use, but thus increase the risk of IA [26]. In addition, Senormancı et al.'s study states that anger significantly predicts the onset of IA and may lead to IA in the form of coping mechanisms if anger is not effectively expressed [27], while Fan et al.'s study states that individuals with IA have an abnormal processing bias for stimuli with angry emotions [28]. From a neurobiological perspective, dysfunction of the dopaminergic pathway is closely related to the development of IA, depression, and anxiety [29]. When individuals are addicted to the Internet, the stimulation it provides prompts the brain to release a large amount of dopamine, allowing them to experience pleasure and satisfaction, and gradually form a dependence on the Internet [30]. Long-term IA will lead to abnormalities in the functioning of the dopaminergic pathway, and this abnormality is also one of the bases for the development of depression, anxiety, and other mood disorders.

In addition, IA in children with malignant tumors was not significantly correlated with pain, peer relationships, physical functioning-mobility, and physical functioning-upper extremity scores in this study. This may be related to the characteristics of the sample in this study; there may be differences in the perception and expression of pain, and in the impact of disease characteristics on physical functioning and mobility among children with different tumor types and treatment stages. The lack of diversity in the sample may have masked potential relationships between IA and these variables.

Limitations and strengths

First, although the multiple linear regression models in this study were generally significant (p < 0.05), their goodness-of-fit was low. This may be attributed to the omission of certain potentially important variables, which limited the models' explanatory power. Future research could optimize these models by incorporating more relevant variables. Second, although the present study demonstrated a positive correlation and significant effect of IA on self-reported indicators of psychological functioning in children with malignant tumors, it was limited by the cross-sectional design and did not reflect a causal relationship. The current evidence is also insufficient to indicate whether IA negatively affects treatment adherence or disease prognosis in this particular population - an area that warrants further exploration in future research. Third, since the sample was exclusively from one medical center, it was potentially limited in representativeness due to geographical and hospital-type constraints. Future investigations should consider expanding the sample size and conducting multi-center, cross-cultural studies to enhance generalizability. Fourth, relying solely on children's self-reports for assessing IA and PROs may introduce subjective bias. To improve the accuracy of future findings, researchers should adopt multi-source data collection methods, such as integrating parental reports and behavioral observations.

The study's major strength lies in its novelty: it transcends previous research focused primarily on healthy populations and, for the first time, examines IA among children with malignant tumors. Through children's self-reported data, it reveals the prevalence of IA within this group and its correlations with psychological functioning indicators. These findings offer parents and clinicians new insights into the behavioral patterns of children with malignant tumors, facilitating comprehensive health management. Clinically, the results enable doctors to elucidate the relationships among addiction, disease, and psychological state to parents, guiding families in establishing healthy digital usage norms. Additionally, the study provides a theoretical foundation for developing tailored IA intervention strategies, aiming to help these children maintain healthy online habits.

## Conclusions

This study reveals the high incidence of IA in children with malignant tumors and the correlation and positive influence of IA on children's psychological function indicators, which provides important clues for the clinical identification of IA and high-risk psychological problems in this population. In the future, clinical staff should pay more attention to the Internet use behavior of children with malignant tumors and focus on observing the children's psychoemotional changes while also monitoring their physical health. Early screening for IA using a standardized scale and developing a personalized Internet behavior management plan in conjunction with a psychological intervention team will reduce the potential threat of IA to children's mental health and promote comprehensive rehabilitation that emphasizes both somatic treatment and psychological care.
